# Dysregulation of the miR‐16‐WWP1 signalling pathway leads to colorectal tumorigenesis

**DOI:** 10.1002/ctm2.709

**Published:** 2022-01-26

**Authors:** Xiaorui Chen, Yi Zhao, Qing Zhu, Yanqing Liu, Yang Luo, Wei Cheng, Bohan Zhang, Kai Wang, Xiaohong Jiang, Rui Liu, Yanbo Wang, Zhen Zhou, Xi Chen

**Affiliations:** ^1^ Nanjing Drum Tower Hospital Center of Molecular Diagnostic and Therapy State Key Laboratory of Pharmaceutical Biotechnology Jiangsu Engineering Research Center for MicroRNA Biology and Biotechnology NJU Advanced Institute of Life Sciences (NAILS) School of Life Sciences Nanjing University Nanjing China; ^2^ State Key Laboratory of Reproductive Medicine Center for Global Health School of Public Health Nanjing Medical University Nanjing China; ^3^ Department of Gastrointestinal Surgery The Affiliated Hospital of Xuzhou Medical University Xuzhou China; ^4^ National Clinical Research Center for Cancer Key Laboratory of Cancer Prevention and Therapy Tianjin's Clinical Research Center for Cancer Tianjin Medical University Cancer Institute and Hospital Tianjin China; ^5^ Research Unit of Extracellular RNA Chinese Academy of Medical Sciences Nanjing China

Dear Editor,

Colorectal cancer (CRC) is one of the leading malignant tumour‐related causes of death worldwide.^1^ WW domain‐containing E3 ubiquitin protein ligase 1 (WWP1) is a ubiquitin protein ligase^2^, ^3^, ^4^ and a potential oncogene in many cancer types.^5^, ^6^, ^7^, ^8^ There is little work to explain the mechanisms by which WWP1 is regulated during tumorigenesis, particularly in CRC.

We first determined the expression pattern of WWP1 by performing immunohistochemistry (IHC) in a commercial tissue microarray containing 90 pairs of CRC and adjacent normal tissue (ANT) specimens (Figure 1A; Table S1). The percentage of WWP1‐positive cells was dramatically higher in CRC specimens compared with ANT specimens (Figure 1B,C). In addition, WWP1 protein levels were positively associated with clinical grades of CRC (Figure 1D). Kaplan–Meier survival analysis showed an increased risk of CRC‐related deaths in patients with higher WWP1 protein levels (Figures 1E). We then confirmed WWP1 protein levels were consistently upregulated in 22 pairs of CRC specimens compared with ANT specimens (Figure 1F,G).

**FIGURE 1:**
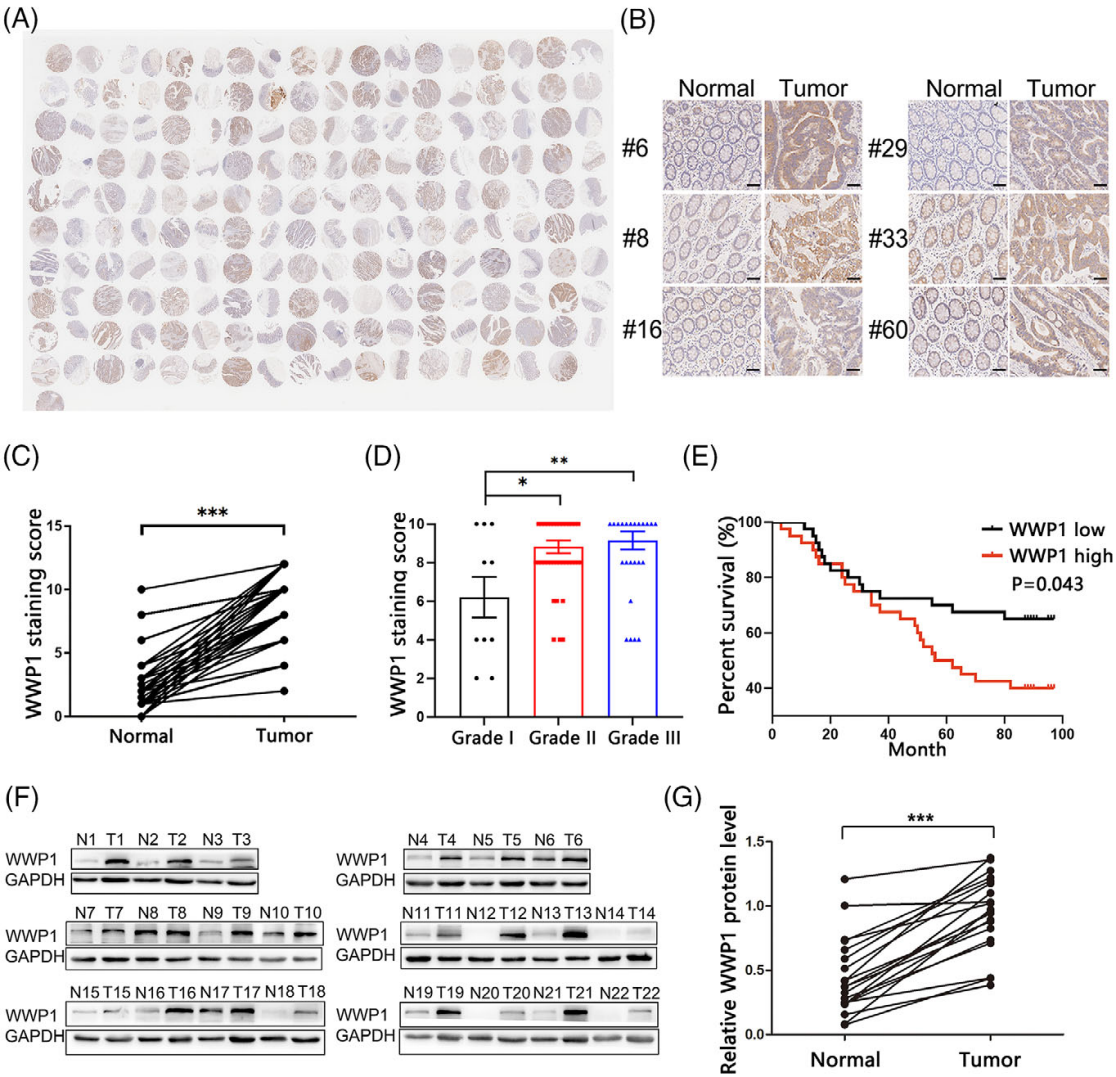
The expression pattern of WWP1 in colorectal cancer (CRC) tissues compared with adjacent normal tissue (ANT) specimens. (A) Image of immunohistochemistry (IHC) staining of WWP1 protein in human CRC tissue microarrays. (B) Representative images of IHC staining of WWP1 protein in CRC tissue microarrays. Scale bar, 100 μm. (C) IHC scores of WWP1 staining in 90 pairs of CRC and ANT specimens (*n* = 90 per group). (D) IHC scores of WWP1 staining in CRC tissue specimens with different grades (*n* = 10, 46 and 34, respectively). (E) Kaplan–Meier curves were generated to analyse the association of WWP1 protein levels in CRC tissue specimens with the overall survival of CRC patients. (F) Western blot analysis of the expression levels of WWP1 protein in 22 pairs of CRC and ANT specimens. (G) Densitometry analysis of the immunoblots from panel F (*n* = 22 per group). Data are shown as the means ± SEMs. *p < .05; **p < .01; ***p < .001

To investigate whether WWP1 could regulate CRC cell proliferation and migration, we transfected a WWP1 overexpression plasmid into SW480 cells. As expected, a dose‐dependent increase in WWP1 protein was observed following transfection with an increasing dose of the WWP1 plasmid; conversely, WWP1 protein expression was inhibited by the transfection of WWP1 siRNA (Figure 2A,B). Both the CCK‐8 and EdU assays revealed that WWP1 siRNA could decrease the cell proliferation rate, while the WWP1 overexpression plasmid augmented it (Figure 2C–E). Likewise, transfection of WWP1 siRNA suppressed cell migration in transwell assay, while transfection of the WWP1 overexpression plasmid stimulated it (Figure 2F,G).

**FIGURE 2:**
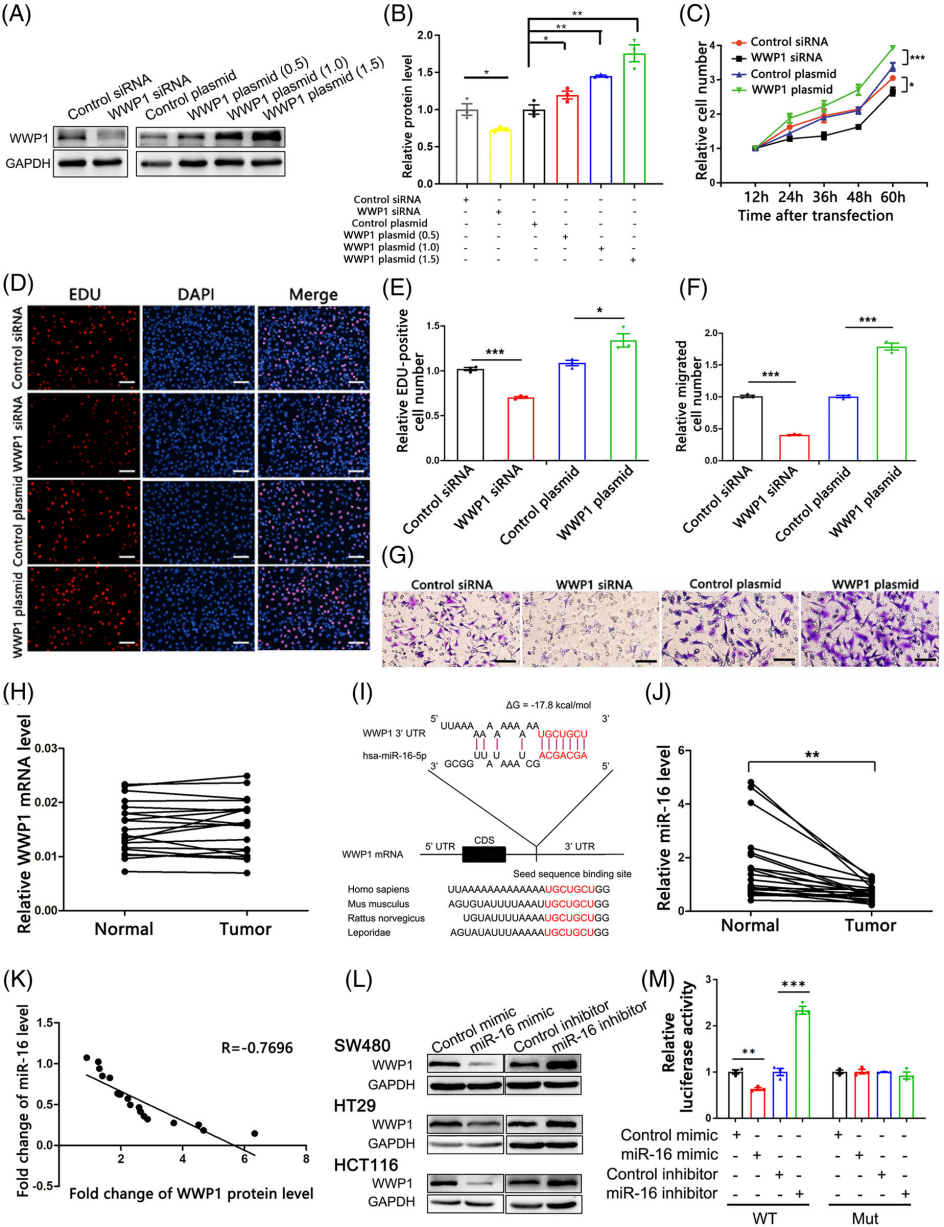
WWP1 functions as an oncogene and is conversely correlated with miR‐16 in colorectal cancer (CRC). (A) Western blot analysis of WWP1 protein levels in SW480 cells transfected with control siRNA, WWP1 siRNA, control plasmid or WWP1 overexpression plasmid (0.5, 1.0 or 1.5 μg). (B) Densitometry analysis of the immunoblots from panel A (*n* = 3 per group). (C) The cell proliferation ability was analysed using the CCK‐8 assay after the transfection of SW480 cells with equal doses of control siRNA, WWP1 siRNA, control plasmid or WWP1 overexpression plasmid (*n* = 3 per group). (D) The EdU proliferation assay was performed 24 h after the transfection of SW480 cells with equal doses of control siRNA, WWP1 siRNA, control plasmid or WWP1 overexpression plasmid. The cells with red fluorescence are in the S phase of mitosis, and the cells with blue fluorescence represent all of the cells. (E) Quantitative analysis of EdU‐positive cells in panel D (*n* = 3 per group). (F) The cell migration ability was analysed using a transwell assay after the transfection of SW480 cells with equal doses of control siRNA, WWP1 siRNA, control plasmid or WWP1 overexpression plasmid. (G) Quantitative analysis of the cells that migrated to the bottom of the transwell membranes (*n* = 3 per group). (H) Quantitative RT‐PCR analysis of the relative expression levels of WWP1 mRNA in CRC and ANT specimens (*n* = 22 per group). (I) Schematic description of the predicted duplexes formed by miR‐16 and the 3′‐UTR of WWP1 mRNA. The predicted free energy value of the hybrid and the seed recognition are indicated, and all nucleotides in this region are highly conserved across species. (J) Quantitative RT‐PCR analysis of the relative expression levels of miR‐16 in CRC and ANT specimens (*n* = 22 per group). (K) Pearson's correlation scatter plot of the fold changes of WWP1 protein and miR‐16 in CRC samples. (L) Western blot analysis of WWP1 protein levels in SW480, HT29 and HCT116 cells transfected with control mimic, miR‐16 mimic, control inhibitor or miR‐16 inhibitor. (M) The relative luciferase activity in 293T transfected with wild type or mutant WWP1 3′‐UTR (*n* = 3 per group). Data are shown as the means ± SEMs. *p < .05; **p < .01; ***p < .001

We explored how the WWP1 protein was regulated in CRC. No significant change in WWP1 mRNA levels and low correlation between WWP1 protein and mRNA levels were detected in the above‐mentioned 22 pairs of CRC samples (Figure 2H; Figure S1), suggesting a posttranscriptional mechanism for the regulation of WWP1. Because miRNAs serve as vital posttranscriptional regulators in various cancers, we speculated that some miRNAs might target WWP1 in CRC. miR‐16 was predicted to be an upstream regulator of WWP1 by two bioinformatic algorithms TargetScan and miRcode. The 3′‐UTR of WWP1 possesses a conserved miR‐16 binding site with a suitable value of minimum free energy of typical miRNA‐target pairs (Figure 2I). Consistently, miR‐16 levels were downregulated in the above‐mentioned 22 pairs of CRC samples (Figure 2J). Pearson's correlation scatter plots confirmed the negative correlation between WWP1 protein and miR‐16 (Figure 2K).

We assessed the alteration of WWP1 protein in three CRC cell lines (SW480, HT29, and HCT116) by transfecting miR‐16 mimic or inhibitor. As expected, the cellular level of miR‐16 altered dramatically after transfection (Figure S2A). Consequently, WWP1 protein expression was markedly reduced by miR‐16 transfection, while miR‐16 inhibitor promoted WWP1 protein expression in CRC cells (Figure 2L; Figure S2B). However, the WWP1 mRNA level was not altered by miR‐16 (Figure S2C). Furthermore, we determined whether miR‐16 directly binds to the WWP1 3′‐UTR via luciferase reporter assay. Luciferase reporter activity was dramatically altered when miR‐16 level was changed while the mutated luciferase reporter was no longer affected by miR‐16 (Figure 2M).

We investigated the cellular phenotypes mediated by the miR‐16‐WWP1 axis. Cell proliferation rate was significantly reduced by miR‐16 mimic, whereas miR‐16 inhibitor markedly boosted proliferation (Figure 3A,C,F). The WWP1 overexpression plasmid was sufficient to rescue the suppression of cell proliferation by miR‐16 (Figure 3B,D,F). Furthermore, miR‐16 mimic markedly decreased cell migration ability, whereas miR‐16 inhibitor increased the number of migrated cells (Figure 3E,G). WWP1 overexpression dramatically attenuated miR‐16‐mediated suppression on cell migration (Figure 3E,G). Likewise, miR‐16 mimic decreased cell invasion; in contrast, miR‐16 inhibition had an opposite effect on invasion (Figure S3). Thus, miR‐16 may regulate proliferation, migration, and invasion in a WWP1‐dependent manner.

**FIGURE 3:**
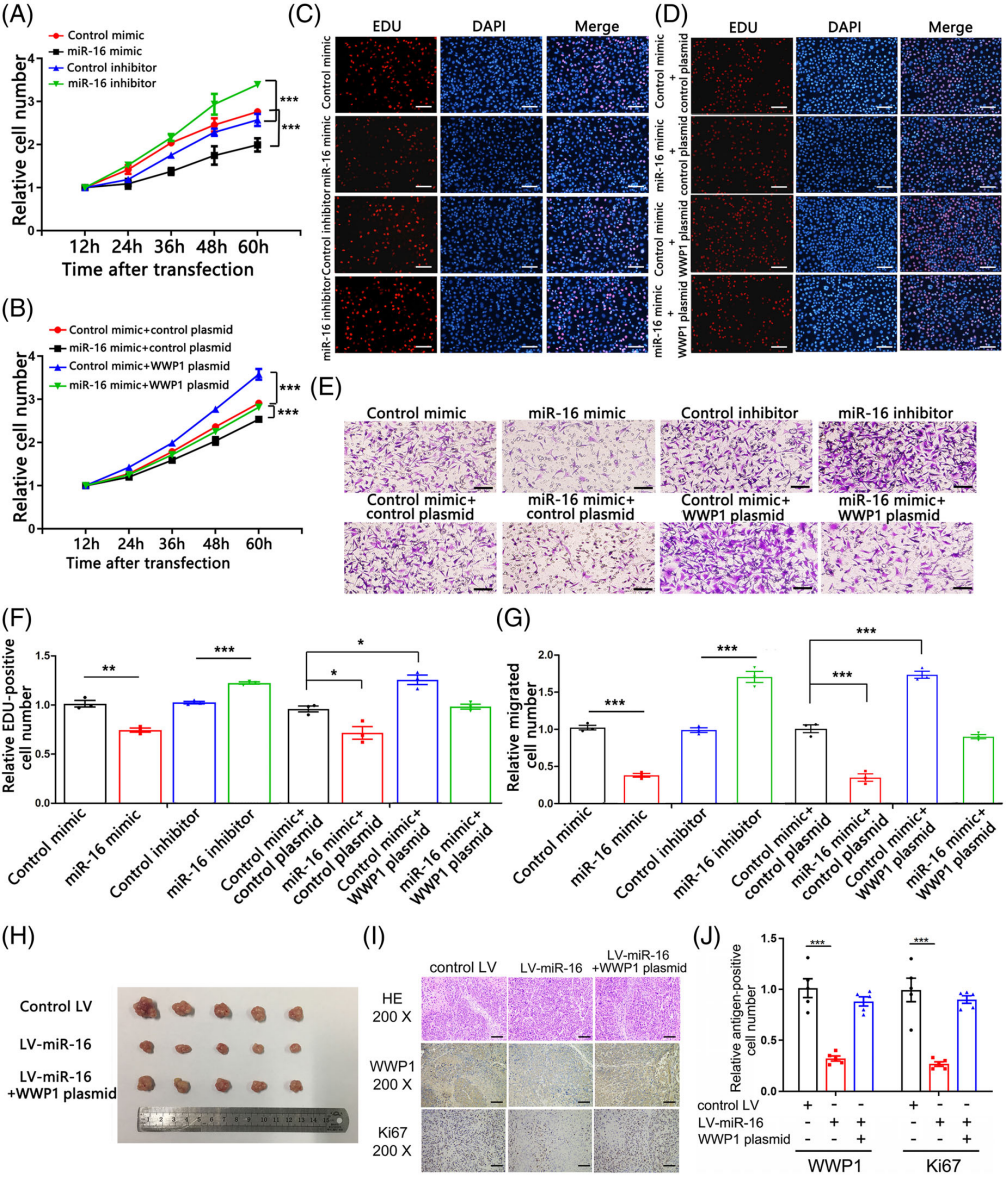
The effects of miR‐16 on WWP1 expression and function in vitro and in vivo. (A) Cell proliferation ability was analysed using the CCK‐8 assay after the transfection of SW480 cells with control mimic, miR‐16 mimic, control inhibitor or miR‐16 inhibitor (*n* = 3 per group). (B) Cell proliferation ability was analysed using the CCK‐8 assay after the cotransfection of SW480 cells with control mimic + control plasmid, miR‐16 mimic + control plasmid, control mimic + WWP1 overexpression plasmid, or miR‐16 mimic + WWP1 overexpression plasmid (*n* = 3 per group). (C) The EdU proliferation assay was performed after the transfection of SW480 cells with control mimic, miR‐16 mimic, control inhibitor or miR‐16 inhibitor. The cells with red fluorescence are in the S phase of mitosis, and the cells with blue fluorescence represent all of the cells. (D) The EdU proliferation assay was performed after the cotransfection with control mimic + control plasmid, miR‐16 mimic + control plasmid, control mimic + WWP1 overexpression plasmid, or miR‐16 mimic + WWP1 overexpression plasmid. The cells with red fluorescence are in the S phase of mitosis, and the cells with blue fluorescence represent all of the cells. (E) Cell migration ability was analysed using a transwell assay after the transfection with control mimic, miR‐16 mimic, control inhibitor or miR‐16 inhibitor or were cotransfected with control mimic + control plasmid, miR‐16 mimic + control plasmid, control mimic + WWP1 overexpression plasmid, or miR‐16 mimic + WWP1 overexpression plasmid. (F) Quantitative analysis of EdU‐positive cells in panels C and D (*n* = 3 per group). (G) Quantitative analysis of the cells that migrated to the bottom of the transwell membranes (*n* = 3 per group). (H) Representative images of the excised tumours. SW480 cells were infected with control LV or a lentivirus overexpressing miR‐16 (LV‐miR‐16) or were cotransfected with LV‐miR‐16 and WWP1 overexpression plasmid. Then, the cells were implanted subcutaneously into four‐week‐old SCID male mice. Tumour growth was evaluated at day 24 after cell implantation. (I) Representative images of H&E‐stained sections of xenografted tumours and representative images of IHC staining for WWP1 and Ki‐67 in xenografted tumours. (J) Quantitative analysis of IHC staining for WWP1 and Ki‐67 (*n* = 5 per group). Data are shown as the means ± SEMs. *p < .05; **p < .01; ***p < .001

We investigated the effects of miR‐16‐WWP1 axis on CRC tumour growth in vivo. An SW480 cell line with stably knockdown of WWP1 by lentivirus (LV‐shWWP1) was implanted into SCID mice. Additionally, SW480 cells infected with control lentivirus or miR‐16 overexpression lentivirus (LV‐miR‐16) or co‐infected with LV‐miR‐16 and the WWP1 overexpression vector were also implanted into SCID mice. Tumour growth was markedly decreased in the LV‐shWWP1 group compared to the control lentivirus group (Figure S4A,B). Slower mitosis, reduced malignancy, lower level of WWP1, and the proliferation marker gene Ki‐67 were observed in the LV‐shWWP1 group by H&E staining and immunohistochemical staining (Figure S4C,D). LV‐miR‐16 clearly reduced tumour growth in vivo, whereas the WWP1 overexpression plasmid attenuated this effect (Figure 3H; Figure S5). Western blotting and immunohistochemical staining showed decreased WWP1 protein level in the LV‐miR‐16 group and rescued expression of WWP1 in the co‐infection group (Figure 3I,J; Figure S6). H&E staining showed that LV‐miR‐16 caused slower mitosis and less malignancy, but the WWP1 overexpression plasmid neutralized this effect (Figure 3I). The Ki‐67 level was decreased in tumours from the LV‐miR‐16 group but was restored in the co‐infection group (Figure 3I,J). These results demonstrated that CRC tumour growth could be inhibited in vivo by miR‐16 via targeting WWP1.

Moreover, we investigated the relationship between miR‐16 and another important regulatory miRNA of WWP1, miR‐452, in CRC cells. miR‐452 could target and repress WWP1 translation in two CRC cell lines (Figure 4A–C). However, miR‐452 and miR‐16 did not display synergism in vitro (Figure 4D–I).

**FIGURE 4:**
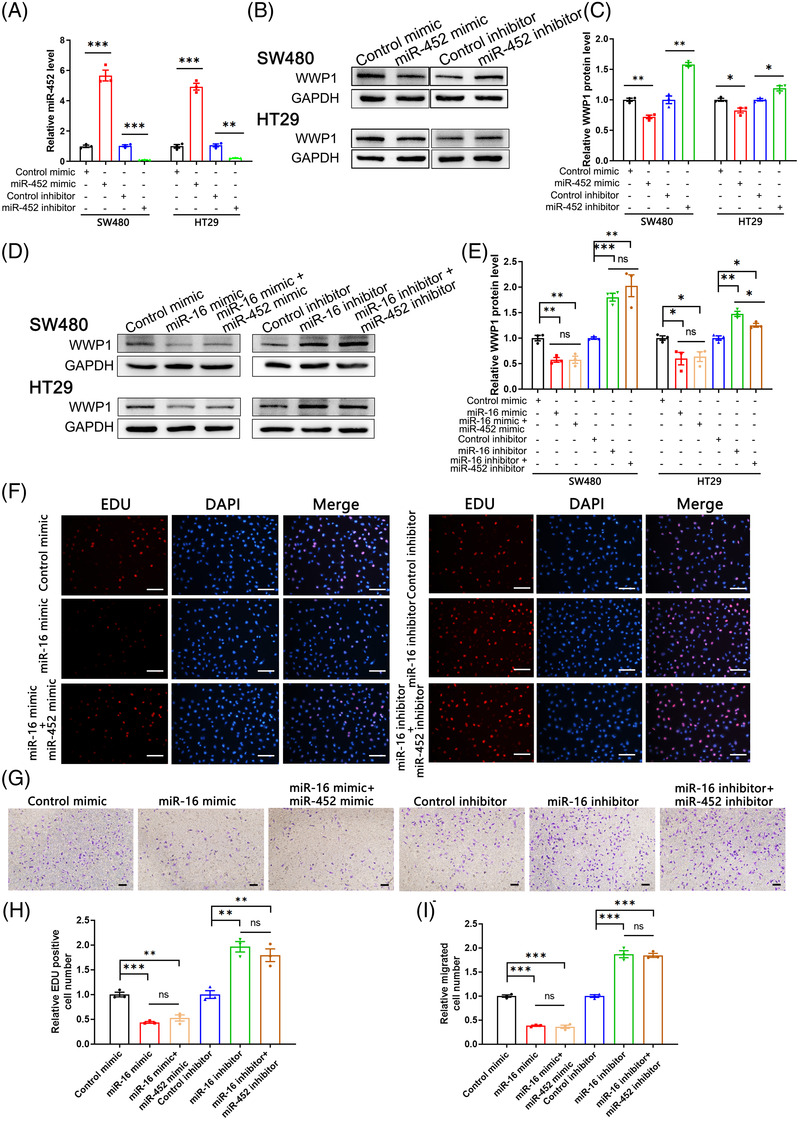
The effects of miR‐452 on WWP1 expression and function in colorectal cancer (CRC) cells. (A) Quantitative RT‐PCR analysis of the relative expression levels of miR‐452 in SW480 and HT29 cells transfected with control mimic, miR‐452 mimic, control inhibitor or miR‐452 inhibitor (*n* = 3 per group). (B) Western blot analysis of WWP1 protein levels in SW480 and HT29 cells transfected with control mimic, miR‐452 mimic, control inhibitor or miR‐452 inhibitor. (C) Densitometry analysis of the immunoblots of WWP1 protein from panel B (*n* = 3 per group). (D) Western blot analysis of WWP1 protein levels in SW480 and HT29 cells transfected with control mimic, miR‐16 mimic, control inhibitor or miR‐16 inhibitor or co‐transfected with miR‐16 mimic + miR‐452 mimic or miR‐16 inhibitor + miR‐452 inhibitor. (E) Densitometry analysis of the immunoblots of WWP1 protein from panel D (*n* = 3 per group). (F) The Edu proliferation assay was performed after transfection with control mimic, miR‐16 mimic, control inhibitor or miR‐16 inhibitor or co‐transfection with miR‐16 mimic + miR‐452 mimic or miR‐16 inhibitor + miR‐452 inhibitor. The cells with red fluorescence are in the S phase of mitosis, and the cells with blue fluorescence represent all of the cells. (G) Cell migration ability was analysed using a transwell assay after transfected with control mimic, miR‐16 mimic, control inhibitor or miR‐16 inhibitor or co‐transfected with miR‐16 mimic + miR‐452 mimic or miR‐16 inhibitor + miR‐452 inhibitor. (H) Quantitative analysis of EdU‐positive cells in panel F (*n* = 3 per group). (I) Quantitative analysis of the cells that migrated to the bottom of the transwell membranes in panel G (*n* = 3 per group). Data are shown as the means ± SEMs. *p < .05; **p < .01; ***p < .001

Overall, we showed that miR‐16 can target WWP1 to suppress CRC tumorigenesis and that downregulation of miR‐16 in CRC abolishes this repression of WWP1, resulting in more malignant tumour features. Our findings also have clinical relevance because targeting WWP1 protein with miR‐16 has the potential to inhibit cellular proliferation and migration for CRC treatment. Indeed, the ubiquitin‐proteasome system has become a popular target for developing therapeutics against tumors.^9^ Our results strongly support this promising strategy because overexpression of miR‐16 or silencing of WWP1 strongly inhibits tumour growth in vivo. Future studies are needed to evaluate the therapeutic efficacy of WWP1 inhibition (e.g., with miR‐16 or siRNA) in disrupting target protein ubiquitination and inducing antitumor activity.

## CONFLICT OF INTEREST

The authors declare no conflict of interest.

## Supporting information

SUPPORTING INFORMATION

SUPPORTING INFORMATION

SUPPORTING INFORMATION
