# Supplementary material for: Dysregulation of the miR‐16‐WWP1 signalling pathway leads to colorectal tumorigenesis
Source: Clin Transl Med. 2022 Jan 26;12(1):e709. doi: 10.1002/ctm2.709 (PMC8792398; doi:10.1002/ctm2.709)
Supplement: Supplementary file 1 — SUPPORTING INFORMATION [file CTM2-12-e709-s001.docx]

**Supplementary Data Set**

**Materials and Methods**

**CRC tissue samples**

Paired CRC cancer and normal noncancerous adjacent tissues were collected from 20 patients who underwent a surgical CRC treatment at Nanjing Drum Tower Hospital (Nanjing, China), which is affiliated with the Medical School of Nanjing University. All protocols regarding the use of CRC samples in this research were approved by the Ethics Committee of Nanjing University. Histological analysis and diagnostic conﬁrmation were performed for both cancer tissues and paired noncancerous adjacent tissues. After surgery, the paired cancer tissues were promptly transferred into containers with liquid nitrogen and frozen at -80 °C. All protocols were performed according to The Code of Ethics of the World Medical Association (statement of Helsinki).

**Tissue microarray and immunohistochemistry**

Tumor tissue microarrays (Outdo Biotech，Shanghai) contain 90 paired CRC cancer together with matched normal noncancerous adjacent tissues and follow-up (range 0–100 months). Immunohistochemistry was performed by two-step method of Dako Envision™ Detection System (DakoCytomation, Glostrup, Denmark). IHC staining score was analyzed via multiplying positive rate score (score of 0, 1, 2, 3 or 4 implied positive areas of 0%, 1–25%, 26–50%, 51–75% or 76–100%, respectively) by staining intensity grade (grade of 0, 1, 2 or 3 represented negative, weak-positive, moderate-positive or strong-positive, respectively). The information of CRC samples is shown in supplemental table.

**Cell culture**

Three human CRC cell lines (SW480, HT29 and HCT116) were obtained from Shanghai Institute of Cell Biology (SIBCB, Shanghai, China). SW480 and HT29 cells were maintained in RPMI 1640 medium (1×) (C1187005500CP, Gibco, California, USA) supplemented with 10% FBS (10099-141, Gibco). Moreover, the HCT116 cell line was maintained in basic DMEM (1×) (C1199550CP, Gibco) supplemented with 10% FBS. All cells were incubated in 5% CO2 at 37 °C in a humidified atmosphere.

**Protein extraction and western blotting**

The proteins of tissues and cell lines were sonicated in RIPA buffer (Beyotime, Shanghai, China), which was supplemented with fresh PMSF (1:100, Beyotime) and fresh protease inhibitor (PI, Thermo, USA) and incubated for 30 minutes on ice. Then, the supernatant was collected after centrifugation for 10 minutes (12000 g, 4 °C). The protein concentration was calculated with a BCA assay kit (Thermo Scientific, USA). Proteins mixed with 5× loading buffer were loaded at the same quantity. Antibodies specific to WWP1 and GAPDH were ordered from Santa Cruz Biotechnology (sc-100679 and sc-25778, respectively; Santa Cruz, CA, USA). Detection was based on a fluorescent secondary antibody that was visualized using the Tanon 5200 Multi detection system (Tanon, Shanghai, China). The data were quantified using ImageJ software (NIH, Bethesda, MD), and the relative protein expression was normalized to the value of GAPDH.

**RNA isolation and quantitative** **RT-PCR**

Total RNA extraction, reverse transcription and TaqMan real-time polymerase chain reaction (PCR) for RNA were performed as described previously. Briefly, total RNA from CRC tissues and three CRC cell lines was extracted using RNAiso Plus reagent according to the product instructions. For mRNA analysis, 1 μg of total RNA was reverse transcribed to cDNA using AMV reverse transcriptase (TaKaRa, Dalian, China) and oligo dT primer (TaKaRa). Quantitative RT-PCR was performed using a SYBR Green PCR kit on an Applied Biosystems 7500 Sequence Detection System. The primer sequences were as follows: WWP1 (sense): TTGAAGGCACGAATGGAATAG and WWP1 (antisense): CAACCTGAGACGGAGATGAAG; GAPDH (sense): CGAGCCACATCGCTCAGACA and GAPDH (antisense): GTGGTGAAGACGCCAGTGGA. For miRNA analysis, 500 ng of total RNA was reverse transcribed to cDNA using AMV reverse transcriptase (TaKaRa) and stem-loop RT primer (Applied Biosystems). Quantitative RT-PCR was performed using a TaqMan PCR kit (000391 and 001973; Shanghai, China). The relative miRNA expression was normalized to U6 snRNA expression.

**WWP1 plasmid and RNA oligo construction**

The WWP1 overexpression plasmid containing the WWP1 ORF and the corresponding empty plasmid, which served as a negative control, were both obtained from GenScript (Nanjing, China). MiRNA mimics, miRNA inhibitors, WWP1 siRNA and negative control RNAs were purchased from GenePharma (Shanghai, China). The WWP1 siRNA and overexpression plasmid were transfected into CRC cell lines by Lipofectamine 2000 (Invitrogen) according to the reagent manual. Total RNA and protein were isolated 48 hours after transfection.

**Luciferase reporter assay**

To test the direct binding of miR-16 to the target gene WWP1, the 3’-UTR of WWP1, which contains the predicted miR-16 target binding site, was inserted into a luciferase reporter plasmid (GenScript, Nanjing, China). To test the binding specificity, sequences that interacted with the miR-16 seed sequence were mutated from AATGTGA to TTACACT, and the synthetic WWP1 3′-UTR mutant fragment was inserted into the same reporter plasmid. The β-galactosidase expression plasmid (β-gal) was obtained from Ambion to evaluate the transfection efficiency. For the detailed luciferase assay, 0.2 μg of β-galactosidase (β-gal) expression plasmid, 0.2 μg of firefly luciferase reporter plasmid, and miR-16 mimic, miR-16 inhibitor or negative control RNAs in equal amounts (50 pmol) were cotransfected into SW480 cells cultured in 24-well plates using Lipofectamine 2000 as per the reagent manual. The β-gal plasmid was used as a transfection control. Twenty-four hours after transfection, we used a luciferase assay kit to measure the cell fluorescence intensity.

**Cell proliferation assay**

Cell Counting Kit-8 (CCK-8) reagent was used to measure the number of cells in the proliferation assay. After transfection of miR-16 mimics, miR-16 inhibitors, siRNAs, overexpression plasmids and corresponding negative controls in SW480 cells, 2 × 10^4^ cells were seeded in each well of 96-well plates. Every 12 hours (until 60 hours) after transfection, 10 μl CCK-8 reagent (CK04-500, Dojindo, Japan) was added to each well to measure the cell proliferation index according to the product instructions. The absorbance value was measured by SpectraMax (Molecular Devices, USA) at 440 nm wavelength.

For the EdU assay, after transfection of miR-16 mimics, miR-16 inhibitors, siRNAs, overexpression plasmids and corresponding negative controls, SW480 cells were seeded in 48-well plates. When the cell density approached 80%, an EdU reagent (RiBoBio, C10310-1, Guangzhou, China) was used to measure the proliferation rate according to the product specification. In this assay DAPI (Beyotime) was used to replace Hoechst provided in the kit. The stained cells were recorded by a BX51 fluorescence microscope (Olympus, Japan).

**Cell migration assay**

To evaluate the cell migration ability, we purchased 24-well plates from Millipore that contain membranes with 8-μm pores. In addition, 60 μl fibronectin (10 μg/mL, Gibco) was added to the bottom of the plates. After transfection of miR-16 mimics, miR-16 inhibitors, siRNAs, overexpression plasmids and corresponding negative controls in SW480 cells, the cells were collected in FBS-free culture medium, and 2 × 10^4^ cells were transferred into the upper chamber. Then, 500 μl of 1640 medium that contained 20% FBS was added to the lower compartment at the same time, and the plates were placed in an incubator in a 5% CO2-humidified atmosphere. After 24 hours, the cells were first fixed by 4% PFA for 30 minutes (room temperature) and then stained with 0.1% crystal violet for 20 minutes (room temperature). The stained cells were observed by a BX51 fluorescence microscope (Olympus, Japan).

**Animal experiments**

Four-week-old male (SCID) mice (*nu/nu*) for use for tumor xenograft studies were obtained from the Model Animal Research Center of Nanjing University (Nanjing, China) and were maintained under specific pathogen-free conditions at Nanjing University. Lentiviruses that can steadily express miR-16 or WWP1 siRNA were purchased from GenePharma. SW480 cell lines were infected with a WWP1 siRNA lentivirus, a miR-16 lentivirus, a control lentivirus, or cotransfected with miR-16 lentivirus and WWP1 overexpression plasmid. After the cell lines were established, we subsequently injected the cells into SCID mice. Each mouse was injected with 3.0 × 10^6^ cells under the left forelimb with an injector. Twenty-four days after injection, the mice were killed. The tumor xenografts were removed, and the weight of the tumors was measured. Parts of the tumors were used for protein and total RNA extraction, and the rest of the tumors were fixed in 4% paraformaldehyde for 24 hours and then processed for H&E and IHC staining for WWP1 and Ki67. All animal studies were approved by the Animal Ethical and Welfare Committee of Nanjing University. The approved ethical number was IACUC-2010001.

**Statistical analysis**

All statistical tests were performed using GraphPad Prism software 8 (San Diego, CA). Data are presented as the means ± SEMs. Normality and equal variances between group samples were assessed using the Shapiro Wilk test and Brown–Forsythe tests, respectively. All the data were normally distributed and had equal variances. All statistical tests were two-tailed, and significance was assigned at P < 0.05 using one-way ANOVA (followed by Tukey's multiple comparisons test) or t-test. The sample sizes and specific statistical tests used in our statistical analyses for each experiment are listed in the figure legends.
