# Supplementary material for: Dysregulation of the miR‐16‐WWP1 signalling pathway leads to colorectal tumorigenesis
Source: Clin Transl Med. 2022 Jan 26;12(1):e709. doi: 10.1002/ctm2.709 (PMC8792398; doi:10.1002/ctm2.709)
Supplement: Supplementary file 3 — SUPPORTING INFORMATION [file CTM2-12-e709-s003.pdf]

# Supplementary Data Set

## Supplementary figure

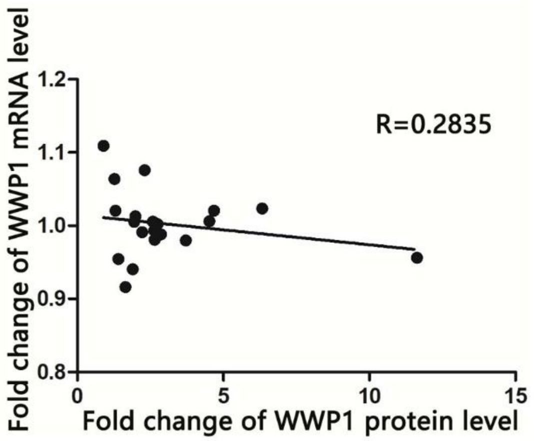


**Figure S1. Pearson's correlation scatter plot of the fold changes of WWP1 protein and mRNA in CRC tissue samples.** n = 22.

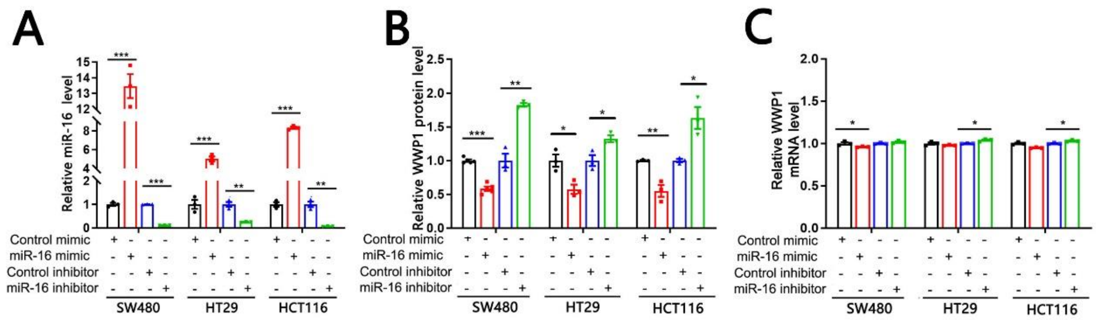


**Figure S2. Overexpression or blockage of miR-16 in CRC cells and the corresponding effects on WWP1 protein and mRNA levels. (A)** Quantitative RT-PCR analysis of the relative expression levels of miR-16 in SW480, HT29 and HCT116 cells transfected with control mimic, miR-16 mimic, control inhibitor or miR-16 inhibitor (n = 3 per group). **(B)** Densitometry analysis of the immunoblots of WWP1 protein from Figure 2L (n = 3 per group). **(C)** Quantitative RT-PCR analysis of the relative expression levels of WWP1 mRNA in SW480, HT29 and HCT116 cells transfected with control mimic, miR-16 mimic, control inhibitor or miR-16 inhibitor (n = 3 per group). Data are shown as the means ± SEMs. *P < 0.05; **P < 0.01; ***P < 0.001.

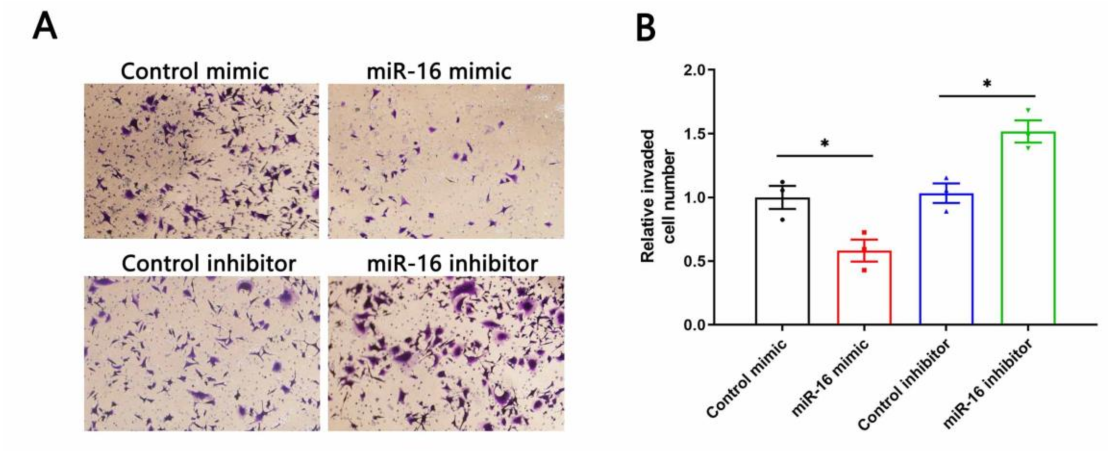


**Figure S3. Effects of miR-16 and WWP1 on CRC cell invasion. (A)** Cell invasion ability was analyzed using a transwell assay after transfected with control mimic, miR-16 mimic, control inhibitor, miR-16 inhibitor. **(B)** Quantitative analysis of the cells that migrated to the bottom of the transwell membranes in panels A (n = 3 per group). Data are shown as the means ± SEMs. $*P < 0.05$.

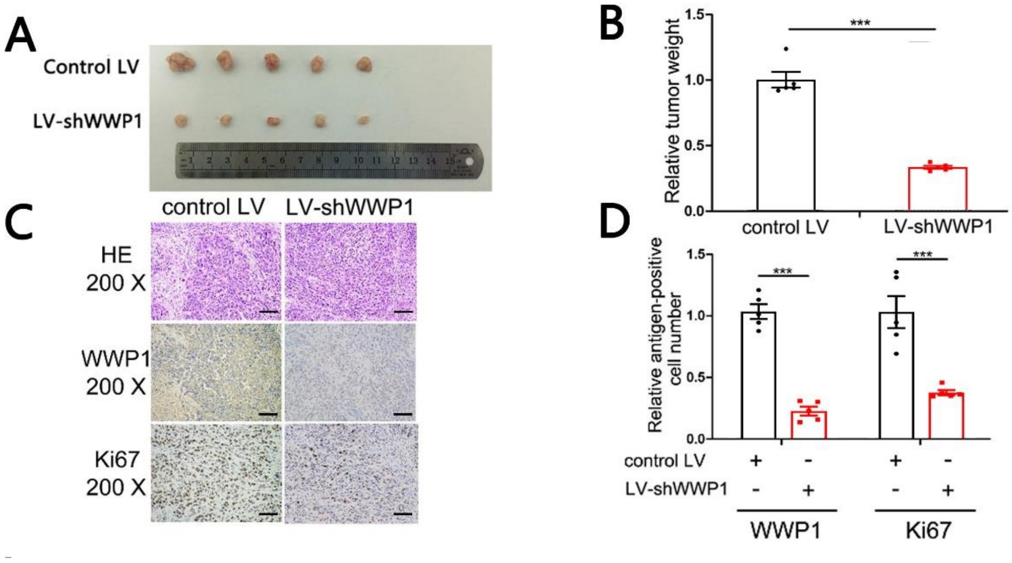


**Figure S4. The effects of WWP1 on CRC tumor growth *in vivo*. (A)** Representative images of the excised tumors. SW480 cells were infected with a control lentivirus (control LV) or a lentivirus overexpressing WWP1 shRNA (LV-shWWP1) and then implanted subcutaneously into four-week-old SCID male mice. Tumor growth was evaluated at day 24 after cell implantation. **(B)** Relative tumor weight (n = 5 per group). **(C)** Representative images of H&E-stained sections of xenografted tumors and representative images of IHC staining for WWP1 and Ki-67 in xenografted tumors. **(D)** Quantitative analysis of IHC staining for WWP1 and Ki-67 (n = 5 per group). Data are shown as the means ± SEMs. ***P < 0.001.

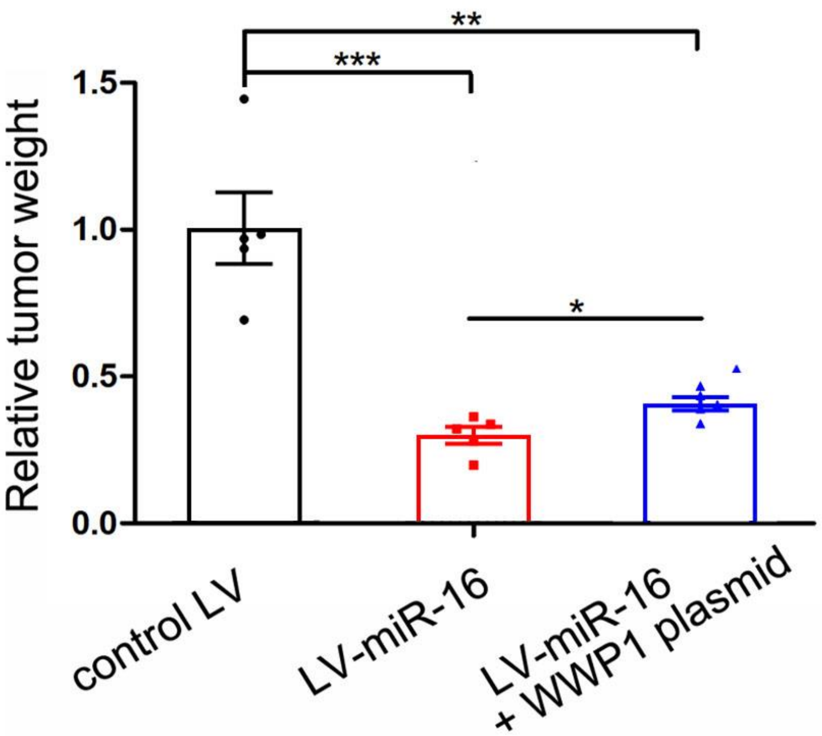


**Figure S5. Relative tumor weight.** n = 5 per group. Data are shown as the means ± SEMs. *P < 0.05; **P < 0.01; ***P < 0.001.

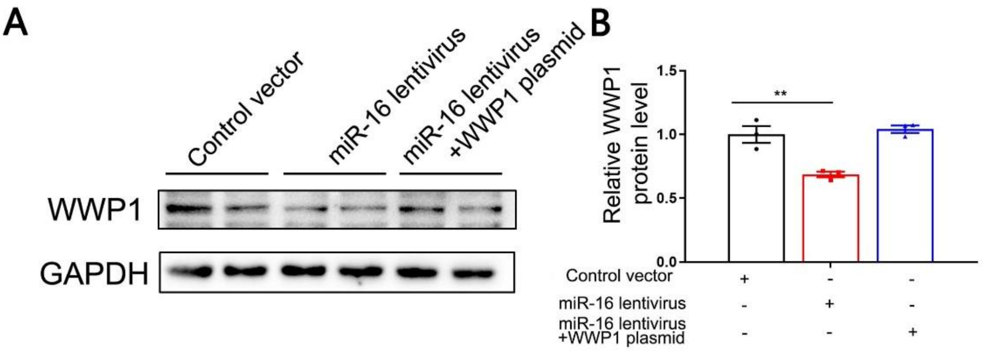


**Figure S6. Western blotting analysis of WWP1 protein levels in xenografted tumors. (A)** Representative western blots. **(B)** Densitometry analysis of the immunoblots from panel A (n = 3 per group). Data are shown as the means ± SEMs. **P < 0.01.

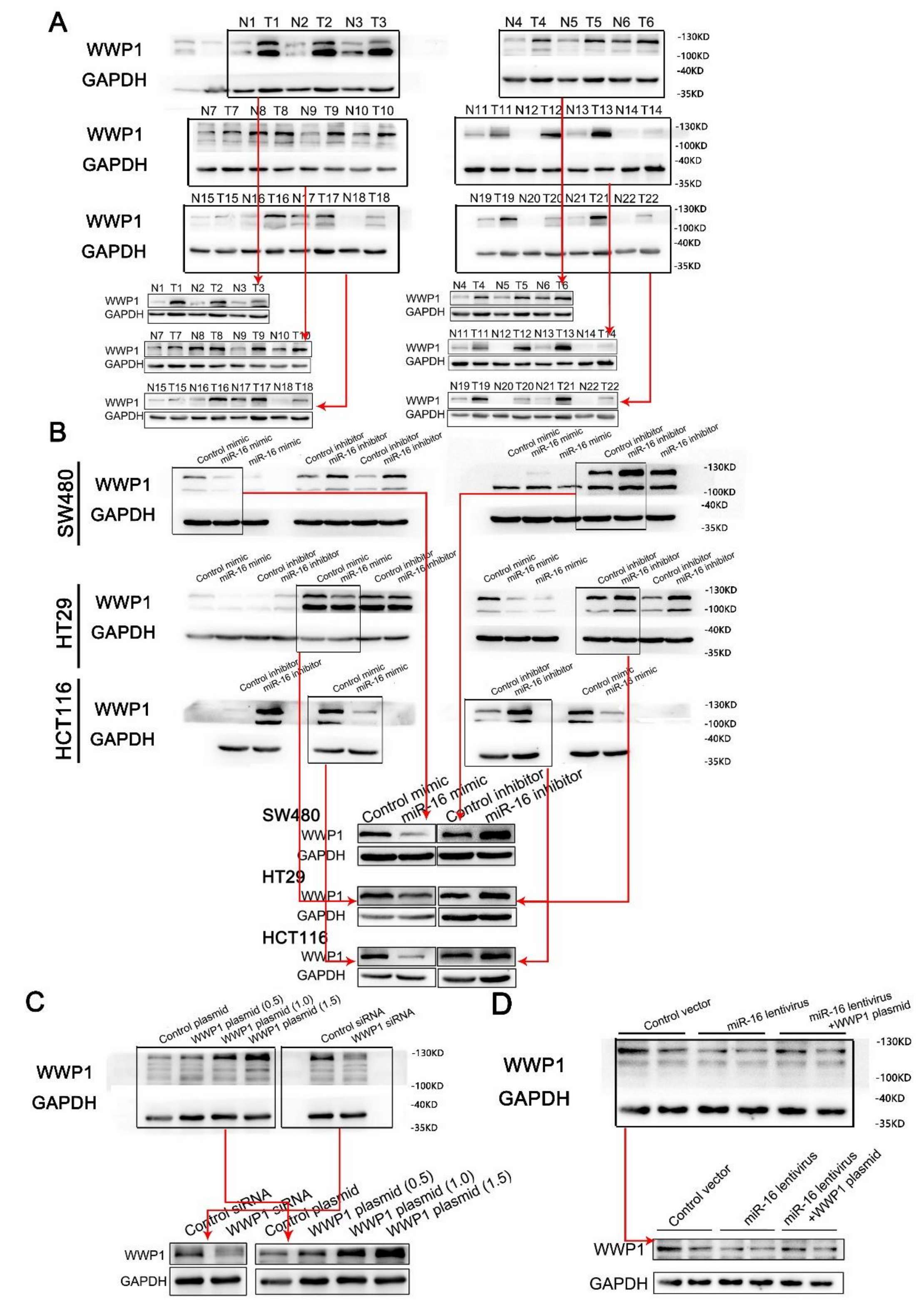


**Figure S7. Original western blot images. (A)** Original western blot images in black frame used for Figure 1F (shown in lower panel). The blots outside the black frame of the upper panels were considered as invalid data due to the degradation of internal control. The red arrow indicates the correspondence between the original western blot images and western blot image of Figure 1F. **(B)**

Original western blot images in black frame used for Figure 2L (shown in lower panel). The blots outside the black frame were duplicate experiments used for statistical analysis. The red arrow indicates the correspondence between the original western blot images and western blot image of Figure 2L. **(C)** Original western blot images used for Figure 2A (shown in lower panel). The red arrow indicates the correspondence between the original western blot images and western blot image of Figure 2A. **(D)** Original western blot images used for Figure S6A (shown in lower panel). The red arrow indicates the correspondence between the original western blot images and western blot image of Figure S6A.
